# The Effect of Various Polyhedral Oligomeric Silsesquioxanes on Viscoelastic, Thermal Properties and Crystallization of Poly(ε-caprolactone) Nanocomposites

**DOI:** 10.3390/polym14235078

**Published:** 2022-11-23

**Authors:** Magdalena Lipińska

**Affiliations:** Institute of Polymer and Dye Technology, Faculty of Chemistry, Lodz University of Technology, Stefanowskiego 16 Street, 90-537 Łódź, Poland; magdalena.lipinska@p.lodz.pl

**Keywords:** poly(ε-caprolactone) PCL, polyhedral oligomeric silsesquioxanes POSS, viscoelastic properties, relaxation analysis, degradation

## Abstract

Polyhedral oligomeric silsesquioxane POSS nanoparticles can be applied as reinforcing additives modifying various properties of biodegradable polymers. The effects of aminopropylisobutyl POSS (amine-POSS), trisilanolisooctyl-POSS (HO-POSS) and glycidyl-POSS (Gly-POSS) on the viscoelastic, thermal properties and crystallization of biodegradable poly(ε-caprolactone) PCL were studied. The analysis of the viscoelastic properties at ambient temperature indicated that aminopropylisobutyl POSS (amine-POSS) and glycidyl-POSS (Gly-POSS) enhanced the dynamic mechanical properties of PCL. The increase in the storage shear modulus *G*′ and loss modulus *G*″ was observed. The plasticizing effect of trisilanolisooctyl POSS (HO-POSS) due to the presence of long isoctyl groups was confirmed. As a result, the crystallization of PCL was facilitated and the degree of crystallinity of *χ_c_* increased up to 50.9%. The damping properties and the values of tan δ for PCL/HO-POSS composition increased from 0.052 to 0.069. The TGA results point out the worsening of the PCL thermal stability, with lower values of T_0.5%_, T_1%_ and T_3%_. Both HO-POSS and Gly-POSS facilitated the relaxation of molten PCL. The presence of Gly-POSS influenced the changes that occurred in the viscoelastic properties of the molten PCL due to the thermo-mechanical degradation of the material; a positive impact was observed.

## 1. Introduction

In modern society, the use of nondegradable low-cost products based on polyethylene (PE), polypropylene (PP) and polystyrene (PS) is growing every day, generating unrecycled plastic waste [1]. Most of the produced plastics are accumulated in landfills [2] or left in the natural environment causing microplastic pollution [3]. This becomes a crucial problem for many countries due to its strong impact on environment and health problems [4,5]. Many intensive research studies are carried out to solve the problems related to plastic waste issues. One essential approach is to use biodegradable materials as an environmentally friendly plastic product, e.g., bottles [6].

Biodegradable polyester materials: poly(lactic acid) PLA, poly(glycolic acid) PGA and polycaprolactone PCL are the class of polymeric materials that show unique properties, biocompatibility and degradation ability, together with excellent mechanical behavior [7,8]. Among biodegradable polyesters, poly(ε-caprolactone) PCL focuses growing attention due to its processing advantages: great electrospinning properties, miscibility with other biodegradable polyesters and thermal stability during processing at a higher temperature [9]. 

Polycaprolactone is a high-strength, crystalline polymer, with a low melting temperature T_m_ in the range 59–64 °C, a glass transition temperature T_g_ of about −60 °C and elasticity at ambient temperature [10]. Its biocompatibility, high material purity and miscibility with other biodegradable materials mean that PCL is used in biomedical applications as: dermal scaffolds [11], bone tissue engineering scaffolds [12,13], stents [14] and electrospun wound dressings [15]. 

The application of PCL as hot-melt adhesive for packaging and bookbinding due to its flexibility and thermo-plasticity properties was reported [16,17]. 

Polycaprolactone can be used as a biodegradable food packaging material, but its poor thermal stability and low melting point restrict its wider application [18]. Here, the blending with natural materials, such as chitosan [18] or bamboo powder [19], gives a good opportunity to create biocomposites with desired properties.

The degradation of polycaprolactone PCL is relatively long due to the structure of PCL and its hydrophobicity preventing water uptake [20]. In environmental waste management, it occurs mostly through the action of fungi, bacteria and algae and extracellular enzymes produced by microorganisms [20]. For biological implants in the physiological environment, two-stage degradation occurs, non-enzymatic hydrolytic degradation and ester linkage breaking followed by enzymatic degradation [12,13]. 

For many application areas the performance of PCL should be improved, especially its mechanical properties, processing stability and resistance to thermo-mechanical degradation. The incorporation of nanoparticles, montmorillonites [21], graphene [22], nanocellulose [23], nanohydroxyapatite [24] enhanced the mechanical strength of PCL. It was shown that different nanoparticles have a positive or negative impact on the degradation of polycaprolactone and other polyesters [25].

Among various nanoparticles, polyhedral oligomeric silsesquioxanes (POSS) are very promising reinforcing additives [26]. Silsesquioxanes POSS are silica cage-like nanometric particles; typically the core is cubic, with a 0.53 nm edge [27]. Their molecular formula is (RSiO_1.5_)_n_, where n can be 8, 10 or 12 [27]. R can be a different functional group attached to the corners of the cage [26,27]. 

Various POSS particles have been incorporated into thermoplastic polyolefins [28,29], polylactide [30], elastomers [31,32], elastomeric blends [33], epoxy resins [34], polysiloxane foams [35], leading to the enhancement of various properties. Polyhedral oligomeric silsesquoixane particles are commercially applied in the cosmetics industry [36]. The low cytotoxicity of POSS allows their application in medicine [37,38]. 

Organic-inorganic hybrid medical biomaterials based on POSS have been developed [39]. Polyhedral oligomeric silsesquoixanes have been used in tissue engineering [40]. Here, the incorporation of POSS into polymeric materials provided the additional enhancement of mechanical properties required for a biological implant. The Young’s modulus of composite was significantly improved [40].

Polyhedral oligomeric silsesquioxanes such as trans-cyclohexanediol isobutyl POSS were incorporated into poly(caprolactone-urea) urethane to obtain a scaffold for dermal replacement. The scaffold consists of two layers, the outer, removable POSS-PCU layer and the inner, biodegradable POSS-PCL layer [11]. 

Electrically conductive nanocomposite material based on POSS-PCL/graphene was studied as a potential material in the field of neural tissue engineering [22]. Trans-cyclohexanediol isobutyl POSS-grafted PCL, due to its biocompatibility and superior mechanical strength, was the ideal constituent material for developing poly(caprolactone-based urea-urethane)(PCL)/graphene hybrid nanocomposite with electrically conductive properties [41]. 

Han et al. [42] reported the compatibilization effect of nanoparticles containing a rigid POSS core and grafted poly(ε-caprolactone) PCL chains on the behavior of poly(lactic acid)/poly(ε-caprolactone) PLLA/PCL blends leading to the better impact strength of the material. 

Lee K.S. et al. [43] found that trisilanolphenyl POSS improved the tensile properties of PCL but the restricted mobility of the PCL chains led to inhibited crystallinity. Oppositely, octavinyl-POSS was found to enhance the isothermal melt crystallization rates of PCL, and the storage modulus of the octavinyl-POSS-PCL nanocomposite increased [44]. A nucleating effect and faster crystallization rate were observed after the incorporation of octaisobutyl-POSS into PCL [45]. 

From the economic point of view, it is important to accomplish the best processing method during nanocomposite preparation. A solution-mixing method followed by film casting [43,44] and the polymerization method [46,47] were used to prepare POSS-PCL nanocomposites. Reactive blending during processing is a key technology for the polymer industry, due to its reduced costs as compared with other methods. Our previous studies [33] showed that the POSS particles containing reactive functional groups can be grafted during the processing of elastomers, resulting in the better morphology and viscoelastic behavior of the material. 

In this work, reactive blending during processing was used to prepare POSS-PCL nanocomposites. The impact of three polyhedral oligomeric silsesquioxane POSS with various functional groups: aminopropylisobutyl-POSS; trisilanolisooctyl-POSS and glycidyl-POSS on the viscoelastic properties of POSS-PCL nanocomposites was studied. To our knowledge, the effects of these POSS on the properties of PCL have not been reported so far in the literature. 

Despite the fact that PCL is more stable during processing compared with other biodegradable polyesters and polylactide PLAs, the challenges associated with the use of biodegradable materials remains similar. Beside the need for mechanical property enhancement, the impact of the nanoparticles on the degradation that occurs in PCLs during the manufacturing process is important. The oscillation and rheological tests of melted PCL at higher temperatures are useful methods to estimate the change in viscoelastic properties resulting from the degradation of the material. With regard to the viscoelastic properties of PCL, it was shown that the presence of POSS particles enhanced not only the dynamic mechanical properties of PCL at ambient temperature, but it also influenced the relaxation behavior of melted PCL and the viscoelastic properties of the thermo-mechanical treated PCL. 

## 2. Materials and Methods

### 2.1. Material and PCL Mixture Preparation

The 3 wt.% of polyhedral oligomeric silsesquioxane POSS with various functional groups was incorporated into ε-polycaprolactone PCL (Sigma-Aldrich Chemistry, St. Louis, MO, USA, CAS 24980-41-4, Mn 70,000–90,000 by GPC). Aminopropylisobutyl POSS (Hybrid Plastics, Hattiesburg, MS, USA, AM0265) with one aminopropyl group and seven isobutyl groups, further denoted as amine-POSS; trisilanolisooctyl-POSS (Hybrid Plastics, Hattiesburg, MS, USA, SO1455), an open-cage POSS with three hydroxyl groups and seven isooctyl groups, further denoted as HO-POSS; a glycidyl-POSS (Hybrid Plastics, Hattiesburg, MS, USA, EP0409) with eight glycidyl groups and epoxy equivalent weight 167, further denoted as Gly-POSS, were used. The melt-mixing method was applied to prepare PCL/POSS composites. For this purpose, the weighed amount of PCL was placed in mixer chamber (Brabender Lab Station Plasti-Corder laboratory mixer with counter-rotating rotors, Brabender GmbH& Co. KG, Duisburg, Germany) and was melted at 100 °C for 10 min. Then, appropriate weighed amount of selected POSS particles (equivalent of 3 wt.%) was added to the melted PCL and mixed (50 r⋅min^−1^ of rotor speed) at 100 °C for 5 min. Then, the compositions were removed from Brabender and cooled. The flat films for rheological and tensile studies were compression-molded using hydraulic press at 200 bar and 100 °C for 5 min. 

### 2.2. Viscoelastic Properties at 25 °C and at Processing Temperature 100 °C

The dynamic viscoelastic properties of PCL-POSS mixtures were studied at 25 °C and at 100 °C in melt state using oscillation rheometer Ares G2 (TA Instruments, New Castle, DE, USA). Plate-plate geometry (diameter 25 mm) was used during measurements. 

Procedures of the tests were: (1) the frequency sweep test at 100 °C, at constant value of oscillation amplitude 1%; (2) the frequency sweep test at 25 °C, at constant value of oscillation amplitude 0.02%; (3) the amplitude sweep test at 25 °C, at constant value of angular frequency 10 rad⋅s^−1^.

The analysis of relaxation of the melted PCL containing various POSS particles was performed based on the frequency sweep test using various relaxation models. 

The Maxwell model [48,49] was applied, and the linear viscoelastic data were used to obtain the discrete relaxation spectrum; the relaxation times *λ_i_*, and values of relaxation modulus *G_i_* were calculated according to below equations.

(1) $$G^{\prime }\left(\omega \right)=\sum_{i=1}^{N}G_{i}\frac{{\left(\omega \lambda_{i}\right)}^{2}}{1+{\left(\omega \lambda_{i}\right)}^{2}}$$

(2) $$G^{\prime \prime }\left(\omega \right)=\sum_{i=1}^{N}G_{i}\frac{\left(\omega \lambda_{i}\right)}{1+{\left(\omega \lambda_{i}\right)}^{2}}$$

Experimentally studied at 100 °C frequency range, the six Maxwell elements were sufficient for recalculation of the values of storage *G*′ and loss shear modulus *G*″ with the level of correlation R^2^ = 0.999.

The continuous relaxation spectrum [50,51,52] was extracted by fitting following model with n terms to oscillation data.

(3) $$G^{\prime }\left(\omega \right)=\int_{-\omega }^{+\omega }H(ln\tau )\;\frac{\omega^{2}\tau^{2}}{1+\omega^{2}\tau^{2}}\;dln\tau$$

(4) $$G^{\prime \prime }\left(\omega \right)=\int_{-\omega }^{+\omega }H\;\left(ln\tau \right)\frac{\omega \tau }{1+\omega^{2}\tau^{2}}\;dln\tau$$

For the numerical computation the spectrum *H*(*lnτ*) was discretized typically in the order of 100 steps. The spectrum represents all the pairs of fitted {*H_i_*,*τ_i_*} parameters.

The fitting of storage shear modulus *G*′ and loss shear modulus *G*″ to the relaxation models was performed using the TRIOS^®^ Software (TRIOS v3 1.5.3696) provided by TA Instruments (New Castle, DE, USA).

Additionally, the zero-shear viscosity *η*_0_ and characteristic mean relaxation times τ_m_ were calculated from viscosity Cole-Cole plots (plots of *η*″ vs. *η*′, where *η*′ is dynamic viscosity and *η*″ is out of phase component of complex viscosity η*) as proposed in the literature [53,54,55,56].

To estimate the changes in the viscoelastic properties caused by the thermo-oxidative and thermo-mechanical degradation, time sweep tests followed by the separate frequency sweep tests were done. The procedures were: (1) for thermo-oxidative degradation-time sweep test: temperature 100 °C, air flow, time 30 min, 60 min, 90 min, 120 min; (2) for thermo-oxidative degradation-time sweep test, temperature 100 °C, air flow, time 30 min, additional mechanical stress, equivalent of 50 s^−1^ shear rate.

### 2.3. The DSC and TGA Analysis

The DSC analysis was performed using DSC1 apparatus (Mettler Toledo LLC, Columbus, OH, USA). All tests were performed under nitrogen atmosphere. The samples were subjected to three heating/cooling/heating steps from −90 °C to 200 °C with the heating rate 10 °C⋅min^−1^. The first heating step was performed to eliminate the thermal history of the sample. The degree of crystallinity *χ_c_* was calculated according to the Equation (6) [12,15]:

(5) $$\chi_{c}=\frac{\Delta H_{m}}{\Delta H_{m}^{0}}\times \frac{100}{w}$$

where Δ*H_m_*—experimental melting enthalpy, $\Delta H_{m}^{0}$—the enthalpy of melting of 100% crystalline PCL (138.5 J g^−1^ [12]), *w*—the weight fraction of material. 

The TGA analysis was performed using TGA/DSC1 (Mettler Toledo LLC, Columbus, OH, USA) analyzer. Samples were heated from 25 °C to 600 °C in an argon with the heating rate 10 °C⋅min^−1^.

## 3. Results and Discussion

### 3.1. The Dynamic Mechanical and Thermal Properties of PCL-POSS Composites at Ambient Temperature

The most popular polyhedral oligomeric silsesquioxane POSS are highly symmetrical silica cage-like structures with a size of approximately 1.5 nm (including the functional R groups) [37,38]. In polymeric systems, the aggregation of POSS occurs naturally, the formed aggregates ranging from 10 to 100 nm in diameter [38,57]. Various POSS particles were incorporated into polymers to improve the dynamic mechanical behavior of the matrix and to generate the reinforcing effect [26]. Here, aminopropylisobutyl POSS (amine-POSS), trisilanolisooctyl-POSS (HO-POSS) and glycidyl-POSS (Gly-POSS) were incorporated into poly(ε-caprolactone) PCL.

**Figure 1:**
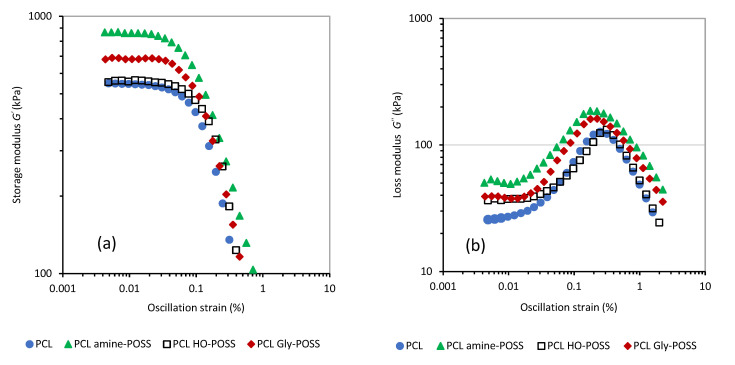
Viscoelastic properties of PCL modified by POSS additives. Storage shear modulus *G*′ (**a**); loss shear modulus *G*″ (**b**) as a function of oscillation strain. Temperature of the measurements 25 °C, angular frequency 10 rad⋅s^−1^.

The oscillation sweep test (Figure 1) at 25 °C confirmed that the selected amine-POSS and Gly-POSS particles enhanced the values of the storage shear modulus *G*′ for the linear viscoelastic region; the values of *G′_LVR_* were as follows: PCL *G′_LVR_* = 545.4 ± 2.4 kPa; PCL amine-POSS *G′_LVR_* = 862.0 ± 3.4 kPa; PCL HO-POSS *G′_LVR_* = 560.9 ± 2.9 kPa; Gly-POSS *G′_LVR_* = 684.5 ± 2.9 kPa. The increase in the loss modulus *G*″ was also observed (Figure 1). It was attributed to the higher dissipation of the energy for the PCL-POSS composites after the incorporation of the amine-POSS and Gly-POSS particles. The incorporation of the POSS particles slightly shifted the cross-points of the *G*′ and *G*″ modulus to higher values of oscillation deformation (%). The cross points *G*′ = *G*″ were as follows: PCL 118.5 kPa at 0.34%; PCL amine-POSS 122.3 kPa at 0.61%; PCL HO-POSS 117.1 kPa at 0.41%; Gly-POSS 131.0 kPa at 0.41%. 

The oscillation sweep test (Figure 1) at 25 °C confirmed that the selected amine-POSS and Gly-POSS particles enhanced the values of the storage shear modulus *G*′ for the linear viscoelastic region; the values of *G′_LVR_* were as follows: PCL *G′_LVR_* = 545.4 ± 2.4 kPa; PCL amine-POSS *G′_LVR_* = 862.0 ± 3.4 kPa; PCL HO-POSS *G′_LVR_* = 560.9 ± 2.9 kPa; Gly-POSS *G′_LVR_* = 684.5 ± 2.9 kPa. The increase in the loss modulus *G*″ was also observed (Figure 1). It was attributed to the higher dissipation of the energy for the PCL-POSS composites after the incorporation of the amine-POSS and Gly-POSS particles. The incorporation of the POSS particles slightly shifted the cross-points of the *G*′ and *G*″ modulus to higher values of oscillation deformation (%). The cross points *G*′ = *G*″ were as follows: PCL 118.5 kPa at 0.34%; PCL amine-POSS 122.3 kPa at 0.61%; PCL HO-POSS 117.1 kPa at 0.41%; Gly-POSS 131.0 kPa at 0.41%. 

The oscillation sweep test (Figure 1) at 25 °C confirmed that the selected amine-POSS and Gly-POSS particles enhanced the values of the storage shear modulus *G*′ for the linear viscoelastic region; the values of *G′_LVR_* were as follows: PCL *G′_LVR_* = 545.4 ± 2.4 kPa; PCL amine-POSS *G′_LVR_* = 862.0 ± 3.4 kPa; PCL HO-POSS *G′_LVR_* = 560.9 ± 2.9 kPa; Gly-POSS *G′_LVR_* = 684.5 ± 2.9 kPa. The increase in the loss modulus *G*″ was also observed (Figure 1). It was attributed to the higher dissipation of the energy for the PCL-POSS composites after the incorporation of the amine-POSS and Gly-POSS particles. The incorporation of the POSS particles slightly shifted the cross-points of the *G*′ and *G*″ modulus to higher values of oscillation deformation (%). The cross points *G*′ = *G*″ were as follows: PCL 118.5 kPa at 0.34%; PCL amine-POSS 122.3 kPa at 0.61%; PCL HO-POSS 117.1 kPa at 0.41%; Gly-POSS 131.0 kPa at 0.41%. 

**Figure 2:**
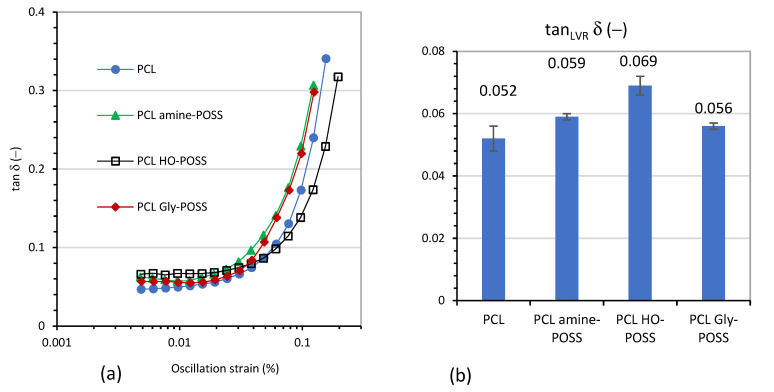
Loss tan δ (−) of PCL modified by POSS additives as a function of oscillation strain (**a**). Values of loss tan δ (−) measured for linear viscoelastic region (LVR) (**b**). Temperature of the measurements 25 °C, angular frequency 10 rad⋅s^−1^.

The values of tan δ at 25 °C as a function of growing deformation (oscillation strain %) are depicted at Figure 2. The incorporation of POSS particles slightly increased the values of tan δ. The HO-POSS most strongly influenced the value of tan δ, as compared with neat PCL, indicating the better damping properties of the material.

**Figure 3:**
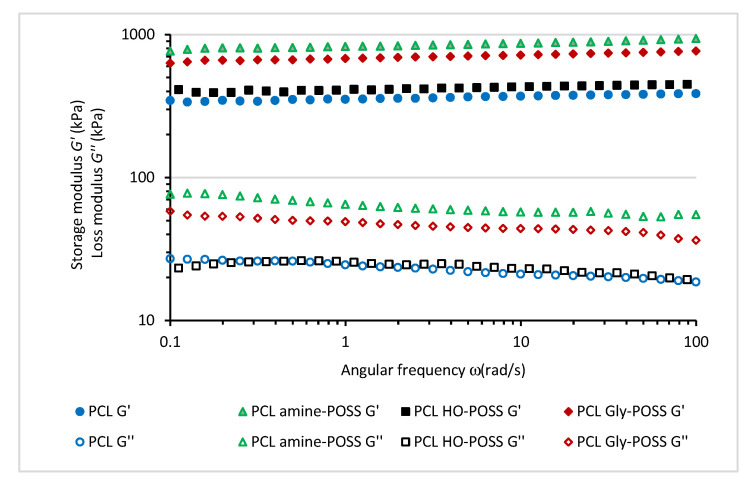
Storage modulus *G′*, loss modulus *G*″ as a function of angular frequency (rad⋅s^−1^) for PCL samples. Temperature of the measurements 25 °C, oscillation strain 0.02 (%).

The frequency sweep test at 25 °C confirmed the reinforcing effect of the selected POSS particles (Figure 3) leading to the enhancement of the PCL dynamic properties. The values of the storage shear modulus *G*′ and loss shear modulus *G*″ after the incorporation of amine-POSS and Gly-POSS increased. No significant effect of HO-POSS on the storage shear modulus *G*′ and loss shear modulus *G*″ of PCL was observed. 

The amine-POSS and Gly-POSS can be applied as reinforcing additive-enhancing dynamic mechanical properties of PCL. The increase in the storage shear modulus *G*′ for filler/polymer systems mainly is a result of three phenomena: the hydrodynamic effect resulting from the fact that the filler is the rigid phase; the interphase filler–polymer interactions causing the occlusion of polymeric chains on the filler surface; and the filler–filler interactions. The nature of the filler, and factors such as: the primary particle size, specific surface area, surface activity and tendency to form aggregate–aggregate associations are important [58]. For the amine-POSS and Gly-POSS particles, two factors determined the reinforcing effect. First, small, nanometric rigid particles, even at lower loading, generated a larger interphase contact area as compared with typical micrometric filler aggregates. Moreover, the functional aminopropyl group or glycidyl groups were able to create filler–polymer interactions with functional groups of polyester leading to the occlusion of polymeric chains at the surface of the POSS nanometric aggregates. The area formed at the filler/polymer interphase by the occlusion of polymeric chains was shielded from deformation and therefore increased the effective filler content. These factors are attributed to the increase in *G*′ observed for the PCL amine-POSS and PCL Gly-POSS samples. The HO-POSS, oppositely to amine-POSS and Gly-POSS, is an open-cage polyhedral oligomeric silsesquioxane that contains three hydroxyl groups attached to the same silicon Si atom as the isooctyl groups. Probably, the formation of the interphase interaction between the hydroxyl groups and polymeric chains was restricted due to steric hindrance and the covering effect of the isooctyl group preventing the penetration of the polymeric chains to the HO-group. As a result, the values of the storage modulus *G*′ for the PCL HO-POSS were lower as compared with the other PCL POSS composites. 

Here, also, the effect of the POSS on the crystallization of PCL was observed, as studied by DSC (Table 1). Both amine-POSS and Gly-POSS decreased the value of the degree of crystallinity *χ_c_*. Oppositely, the incorporation of HO-POSS enhanced the degree of crystallinity. The longer isooctyl groups present in HO-POSS had a plasticizing effect on the melted PCL, as analyzed further, and this probably facilitated crystallization.

Other authors [44,45] also reported the various influences of POSS particles on the crystallization of PCL due to the structure of POSS [43,44,45]. The presence of POSS may provide heterogenous nucleation sites increasing the degree of crystallinity, while the aggregates of POSS may restrict the growth of large crystals of PCL [45]. 

The glass transition temperature T_g_ of PCL was shifted to higher values of temperature after the incorporation of selected POSS confirming the restricted mobility of the polymeric chains in the presence of POSS. Other authors [43] also observed this effect for trisilanolphenyl-POSS. 

The POSS less meaningfully changed the melting temperature T_m_ and crystallization temperature T_c_ of PCL. The DCS plots for the studied samples are compiled in Supplementary Materials (Figures S1–S4).

**Figure 4:**
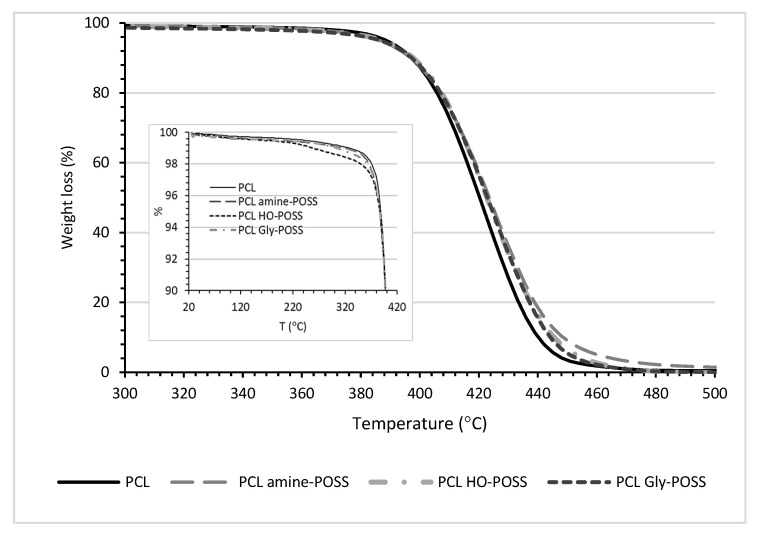
TGA plots for PCL POSS samples.

The TGA studies (Table 2, Figure 4) showed that POSS particles influenced the thermal behavior of the sample. It was observed that the 0.5%, 1% and 3% sample weight losses were measured at a lower temperature for the samples containing POSS particles. Particularly, a worsening of thermal stability was observed for PCL HO-POSS. Similarly, the temperature of the 5% weight loss as measured for neat HO-POSS was also lower (210 °C), as compared with other POSS: for Gly-POSS, 365 °C, and for amine-POSS, 221 °C, respectively.

The HO-POSS, oppositely to amine-POSS and Gly-POSS, is an open-cage polyhedral oligomeric silsesquioxane. Bautista Y. et al. [59] showed the slight positive impact of open-cage oligomeric silsesquioxanes on the thermal stability of polyester due to the shift of the temperature at 50% weight loss. Here, also, a slight shift of T_50%_ for all PCL POSS compositions from 421 to 424 °C was observed. The authors [1] also analyzed the influence of the POSS particles on the mechanism of the thermal degradation of polyester. In fact, the thermal degradation of PCL and the hydrolysis of this polymer are affected by the water up-take [20]. Comparing polylactide PLA with poly(ε-caprolactone) PCL, the water uptake is more difficult due to the higher hydrophobicity of this polymer [20]. The presence of POSS, especially those with polar groups with a high affinity to water molecules, can increase the water up-take. The presence of water molecules can be disadvantage during processing, leading to changes in the properties of the material, but TGA plots showed that the process of 0.5% weight loss occurred at a higher temperature than the processing temperature of 100 °C. However, the presence of the water molecules could be the reason that the process of the hydrolysis of the material started at a lower temperature as compared with the neat PCL. Probably, the presence of POSS facilitated the diffusion of water throughout the material. This can be an advantage, because low water up-take is one of factors influencing the slow degradation rate of PCL during compositing. 

### 3.2. The Viscoelastic Properties, Relaxation and Degradation of PCL-POSS at Temperature of 100 °C

Dynamic oscillatory tests are a very sensitive method to indicate the changes occurring in the properties of melted polymers due to the incorporation of nanoparticles or due to the thermal, thermo-mechanical or thermo-oxidative degradation of polymeric material. Various POSS were found to affect the rheological behavior of the processed polymers, especially the viscosity. The incorporation of 3 wt.% of HO-POSS slightly decreased the complex viscosity η* at 100 °C. The opposite effect was observed for amine-POSS (Supplementary Materials Figure S5). The values of complex viscosity η* at 0.1 rad⋅s^−1^ were: PCL η*_0.1rad/s_ = 2470 Pa⋅s; PCL amine-POSS η*_0.1rad/s_ = 2567 Pa⋅s; PCL HO-POSS η*_0.1rad/s_ = 2167 Pa⋅s; PCL Gly-POSS η*_0.1rad/s_ = 2456 Pa⋅s, respectively. 

**Figure 5:**
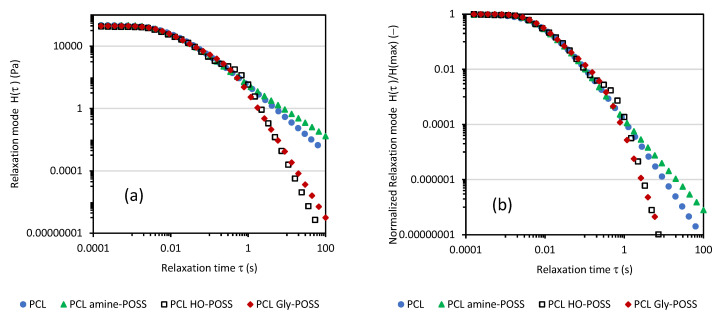
Continuous relaxation spectra for PCL samples modified by POSS (**a**). Normalized relaxation spectra for PCL samples modified by POSS (**b**).

Based on the storage shear modulus *G*′ and loss shear modulus *G*″ measured as a function of angular frequency ω at 100 °C (Supplementary Materials Figure S5), the continuous relaxation spectra were calculated according to Equations (3) and (4). The calculated values of *H(τ)* are collected in Figure 5.

Both HO-POSS and Gly-POSS facilitated the relaxation of melted PCL. Oppositely, longer relaxation times were observed for amine-POSS PCL indicating the lower mobility of the PCL chains, probably due to interactions between the aminopropyl group of POSS and PCL chains. The observed facilitation of the relaxation of the melted PCL in the presence of HO-POSS and Gly-POSS can be an advantage during processing when high deformation (shear rate) on the material is applied.

The poly(ε-caprolactone) PCL belongs to a polyester that shows biodegradability. The ability to degrade under some conditions can be an ecological advantage facilitating the protection of the environment and waste management. From another point of view, polymer material should be stable during processing and not change viscoelastic properties. Frequency sweep tests were done for the melted PCL samples before and after thermal treatment at 100 °C for 30, 60, 90, 120 min. The melted PCL material was exposed only to the temperature and the oxygen present in the air; no mechanical stress was applied. The thermo-oxidative degradation of the melted PCL, up to 120 min, slightly changed the viscoelastic properties of the material. No significant changes in the values of the storage shear modulus *G*′ and loss shear modulus *G*″ were observed. However, the cross points *G*′ = *G*″ determined from the frequency sweep tests were shifted to different values of angular frequency ω (Supplementary Materials Table S1), as the time of the thermo-oxidative degradation of the melted PCL was longer.

**Figure 6:**
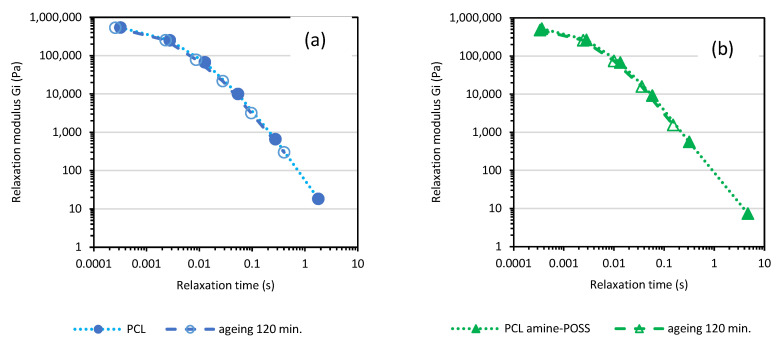
Calculated based on frequency sweep test discrete relaxation Maxwell’s spectra for PCL (**a**) and PCL modified by amine-POSS (**b**).

**Figure 7:**
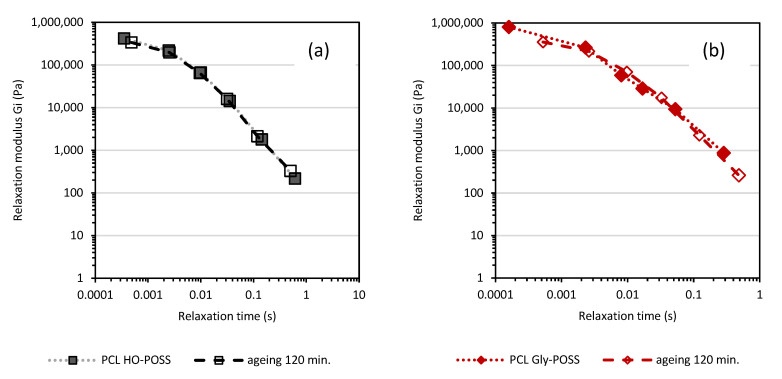
Discrete relaxation Maxwell’s spectra for PCL HO-POSS (**a**) and PCL Gly-POSS (**b**).

The calculated relaxation spectra before and after thermal treatment at 100 °C can give useful information about the change in materials due to thermo-oxidative degradation. The calculations according to the Equations (1) and (2), Maxwell’s discrete relaxation spectra for the samples before and after thermo-oxidative treatment for 120 min at 100°, are shown in Figure 6 and Figure 7. The calculated values of relaxation modulus G_i_ and the relaxation times *λ_i_* after 30, 60, 90, 120 min of thermo-oxidative treatment are compiled in Tables S2–S5 (Supplementary Materials Tables S2–S5). Thermo-oxidative degradation over 120 min. at 100 °C reduced the values of the relaxation times λ calculated for every Maxwell element for both PCL and PCL amine-POSS indicating the changes in the structure of the melted PCL.

Less significant changes in the values of the relaxation modulus G_i_ and the relaxation times *λ_i_*, recalculated based on similar angular frequency range, were observed for the PCL HO-POSS and PCL Gly-POSS composites indicating that the presence of both POSS-stabilized materials during thermo-oxidative treatment as compared with neat PCL (Figure 7). 

The *R.M.I.* (Relative Modification Index), calculated based on the measurement of complex viscosity at constant temperature, can be used to compare the influence of various additives on the degradation of the material [48]. The *R.M.I.* is calculated according to the below equation (Equation (6)), using the values of complex viscosity η* determined from the viscosity plateau of the η* versus angular frequency ω plots for the samples before and after thermo-oxidative ageing over 30, 60, 90, 120 min at 100 °C in the presence of the oxygen from air.

(6) $$R.M.I.=\frac{\eta_{time=0}}{\eta_{time}}$$

**Figure 8:**
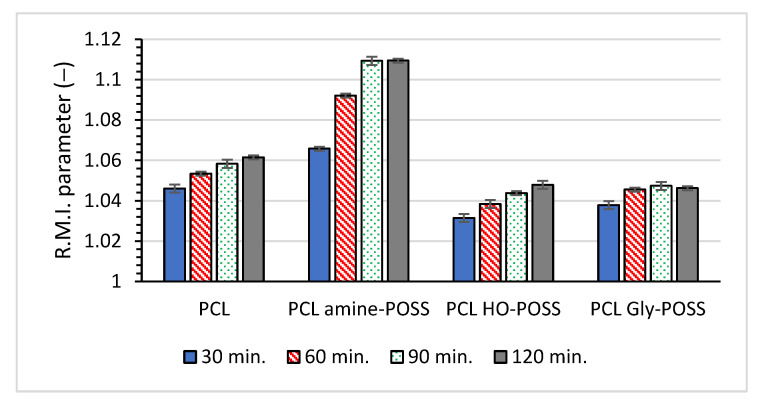
Calculated from the complex viscosity plot *R.M.I.* parameters for PCL samples after thermo-oxidative treatment.

The values of the *R.M.I.* parameter higher than 1 for samples after thermo-oxidative ageing indicated the decrease in the viscosity of the sample due to chain cleavage occurring during degradation. No meaningful change in the values of *R.M.I.* after thermo-oxidative treatment for all PCL samples was observed (Figure 8). It confirmed that the thermo-oxidative treatment of the PCL did not affect strongly the viscous properties of the material. However, the presence of POSS influenced the values of the *R.M.I.* parameter showing that both HO-POSS and Gly-POSS slightly stabilized the material as compared with neat PCL and PCL amine-POSS. The observations from the relaxation studies are in agreement with the calculated values of the *R.M.I.* parameter for the PCL samples. 

During processing, melted polymeric material is exposed not only to temperature and oxygen but also to mechanical stress. To estimate the change in the viscoelastic properties of the PCL POSS composites occurring due to thermo-mechanical degradation at 100 °C and additionally the mechanical stress, the equivalent of 50 s^−1^ shear rate, over 30 min, was applied. 

**Figure 9:**
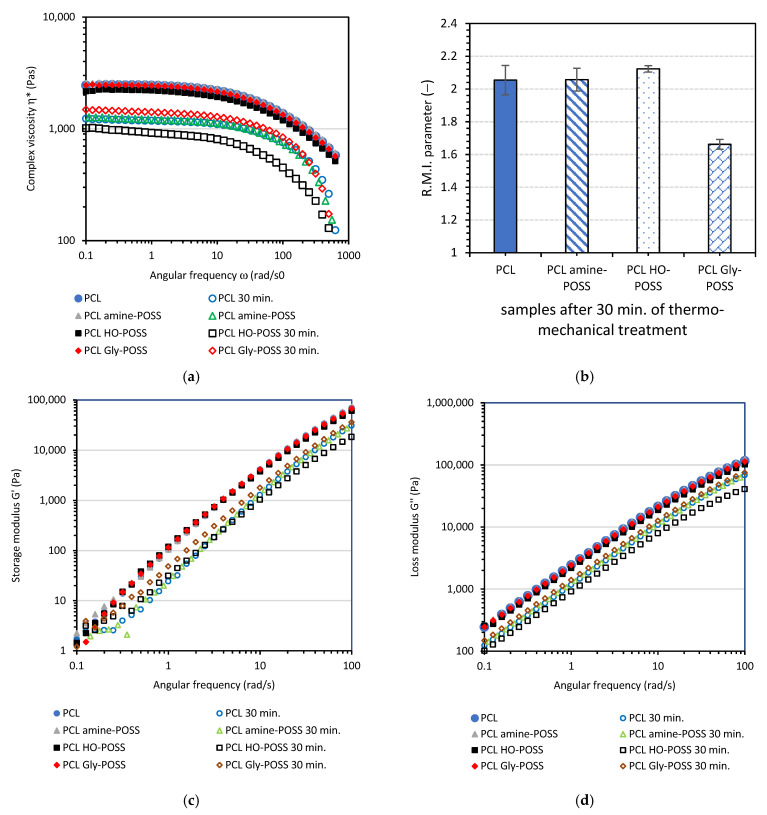
Viscoelastic properties at 100 °C of PCL modified by the addition of POSS particles, complex viscosity η* (**a**), calculated values of *R.M.I.* parameter (**b**), storage shear modulus *G*′ (**c**), loss shear modulus *G*″ (**d**), for PCL samples before and after thermo-mechanical ageing (condition of ageing: temperature 100 °C, mechanical stress equivalent of 50 s^−1^ shear rate, time 30 min).

The viscoelastic properties of the samples before and after thermo-mechanical treatment were studied. Figure 9 shows the values of complex viscosity η*, storage shear modulus *G′*, loss shear modulus *G*″ as a function of angular frequency ω and the calculated values of the *R.M.I.* parameter for the PCL samples before and after thermo-mechanical treatment (30 min at 100 °C, applied mechanical stress, equivalent of 50 s^−1^ shear rate).

As expected, the degradation of the PCL samples after the application of mechanical stress occurred to a higher extent, and the decrease in complex viscosity η* was stronger. The reduction of complex viscosity Δη* at 0.1 rad⋅s^−1^ was calculated according to Equation (7):

(7) $$\Delta \eta^{*}=\eta_{before\;ageing}^{*}-\eta_{after\;thermo-mechanical\;ageing}^{*}$$

The calculated values of Δη* were as follows: Δη* = 1256 Pas for PCL; Δη* = 1320 Pas for PCL amine-POSS; Δη* = 1147 Pas for PCL HO-POSS; Δη* = 984 Pas for PCL Gly-POSS.

Further, the thermo-mechanical degradation caused the decrease in the storage shear modulus *G*′ and loss shear modulus *G*″ (Figure 9). Here, the positive influence of the Gly-POSS on the material is also observed. The decrease in the storage shear modulus *G*′ measured at 100 rad⋅s^−1^ for PCL Gly-POSS was 31.4 kPa as compared with 38.9 kPa for neat PCL, 41.8 kPa for PCL amine-POSS and 42.3 kPa for PCL HO-POSS. The calculated values of the *R.M.I.* parameter also showed the higher stability of the PCL Gly-POSS composition during thermo-mechanical treatment.

**Figure 10:**
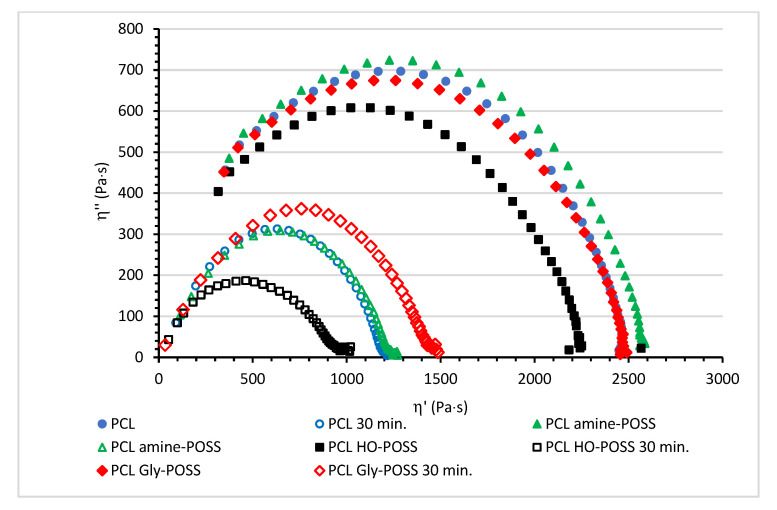
The Cole-Cole plots for PCL and PCL modified POSS at 100 °C; *η*′—dynamic viscosity (Pa⋅s), *η*″—out of phase component of complex viscosity η* (Pa⋅s).

The viscosity Cole-Cole plots, plots of *η*″ (out of the phase component of the complex viscosity η*) versus *η*′ (dynamic viscosity), are a useful tool to study the influence of the additives on the rheological behavior and the relaxation of the melted polymer. Some rheological parameters that can be derived from the Cole-Cole plots are the zero-shear viscosity *η*_0_ and characteristic relaxation time [53,54,55,56]. The Cole-Cole plots of the PCL and POSS-PCL samples before and after thermo-mechanical degradation over 30 min at 100 °C are depicted in Figure 10.

All PCL compositions showed a semicircular shape of Cole-Cole plots, typical for homopolymers with monomolecular weight distribution. The incorporation of various POSS particles shifted the maximum of the arc as compared with the neat PCL. The relaxation behavior of the melted PCL was affected by the presence of POSS. The maximum of the semicircular arc was meaningfully shifted after the addition of the HO-POSS. It was observed at lower values of dynamic viscosity *η*’ and higher value of angular frequency ω. The relaxation of the PCL HO-POSS was facilitated. Oppositely, the maximum of the semicircular arc for PCL amine POSS was shifted to a higher value of dynamic viscosity (lower value of angular frequency). The presence of amine-POSS disturbed the relaxation as compared with the neat PCL. 

The calculated values of mean relaxation time τ_m_ (s) were: PCL τ_m_ = 0.0124 s; PCL amine-POSS τ_m_ = 0.0126 s; PCL HO-POSS τ_m_ = 0.0111 s; PCL Gly-POSS τ_m_ = 0.0124 s. 

Moreover, the plasticizing effect of HO-POSS was observed. As it was described previously, the hydroxyl groups are covered by and attached to the same silicon atom as isooctyl groups. These isooctyl groups enhance the plasticization of PCL. The plasticizing effect of various POSS particles was observed by some authors [60] for other biodegradable polyester poly(lactic acid) PLAs. Oppositely, amine-POSS caused the increase in zero-shear viscosity *η*_0_ as compared with neat PCL or PCL or Gly-POSS samples. The calculated values of zero-shear viscosity *η*_0_ (Pas) were: PCL *η*_0_ = 2442 Pas; PCL amine-POSS *η*_0_ = 2587 Pas; PCL HO-POSS *η*_0_ = 2241 Pas; PCL Gly-POSS *η*_0_ = 2490 Pas.

Thermo-mechanical degradation over 30 min decreased the values of dynamic viscosity *η*′ and the out of phase component of complex viscosity *η*″ for all PCL compositions due to PCL degradation and chain cleavage. The calculated values of zero-shear viscosity for samples after thermo-mechanical treatment were: PCL_degraded_ *η*_0_ = 1217 Pas; PCL_degraded_ amine-POSS *η*_0_ = 1246 Pas; PCL_degraded_ HO-POSS *η*_0_ = 1015 Pas; PCL_degraded_ Gly-POSS *η*_0_ = 1485 Pas. The Cole-Cole plots were significantly shifted to the left corner confirming the facilitated relaxation of the degraded materials as compared with these before the thermo-mechanical treatment. The calculated mean relaxation times τ_m_ for the samples after the thermo-mechanical treatment were: PCL_degraded_ τ_m_ = 0.0071 s; PCL_degraded_ amine-POSS τ_m_ = 0.0089 s; PCL_degraded_ HO-POSS τ_m_ = 0.0079 s; PCL_degraded_ Gly-POSS τ_m_ = 0.0113 s. 

**Figure 11:**
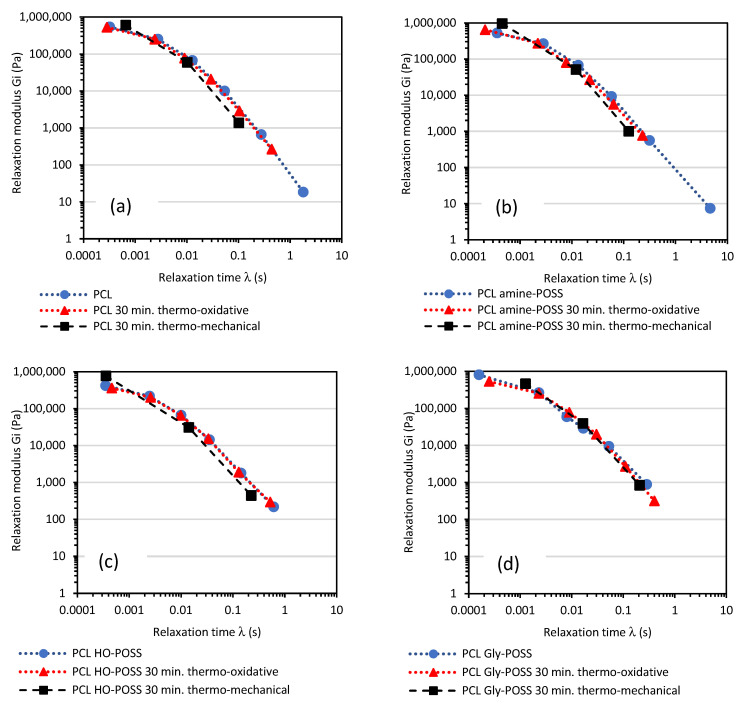
Discrete relaxation Maxwell’s spectra for PCL (**a**) and PCL modified by POSS (**b**–**d**), samples before and after thermo-oxidative ageing (100 °C, oxygen) or thermo-mechanical ageing (100 °C, oxygen, mechanical stress equivalent of 50 s^−1^ shear rate) over 30 min.

For all the PCL samples, thermo-mechanical degradation led to changes in the Maxwell relaxation spectrum (Figure 11). It confirms the observation from the Cole-Cole plots analysis; the relaxation was facilitated due to lower viscosity resulting from PCL chain cleavage. The discrete relaxation Maxwell’s plots of PCL, PCL amine-POSS and PLA HO-POSS were shifted towards lower values of relaxation times. Here, the influence of mechanical stress applied during ageing is evident. The changes in relaxation Maxwell’s spectra for samples exposed only for temperature of 100 °C and oxygen present in the air over 30 min were less visible. The POSS additives, such as HO-POSS and Gly-POSS, were able to stabilize the viscoelastic properties of the melted PCL exposed at 100 °C and in oxygen over 30 min. As a result, no significant changes in the calculated Maxwell’s relaxation spectra were observed. Moreover, no significant shift of discrete Maxwell’s relaxation spectra towards lower values of relaxation times was observed for PCL Gly-POSS. However, the relaxation modulus G_i_ decreased. The degradation of the PCL was taking place as a result of the exposure to temperature, oxygen and mechanical stress. However, the changes in the viscoelastic properties of the PCL occurred to a lesser extent in the presence of Gly-POSS in the composition.

## 4. Conclusions

Aminopropylisobutyl POSS (amine-POSS) and glycidyl POSS (Gly-POSS) increased the values of the storage shear modulus *G*′ and the loss shear modulus *G*″ of poly(ε-caprolactone), as measured in the function of the oscillation strain at 25 °C. The reinforcing effect was attributed to the stronger hydrodynamic effect and interphase interactions of POSS functional groups with polymeric chains. 

Trisilanolisooctyl POSS (HO-POSS) decreased the values of dynamic viscosity *η*′ at 100 °C due to plasticizing effect. The plasticizing effect of HO-POSS was responsible for the facilitation of the crystallization leading to higher values of degree of crystallinity *χ_c_*. Oppositely, the presence of amine-POSS and Gly-POSS slightly reduced the degree of crystallinity *χ_c_* of the PCL as compared with neat PCL. 

The calculated continuous relaxation spectra showed that both HO-POSS and Gly-POSS facilitated the relaxation of melted PCL. Calculations from the Cole-Cole plots’ mean relaxation time τ also confirmed the facilitated relaxation of the melted PCL HO-POSS composition. Longer relaxation times were observed for amine-POSS/PCL composition. 

Thermo-oxidative degradation at 100 °C for up to 120 min slightly changed the viscoelastic properties of PCL. However, the lower values of the calculated *R.M.I.* parameter suggested the higher stability of the melted PCL in the presence of HO-POSS and Gly-POSS. 

The thermo-mechanical degradation of PCL resulted in a decrease in complex viscosity η*, storage modulus *G*′ and loss modulus *G*″. The Maxwell’s relaxation spectra shifted to lower values of relaxation times λ, indicating that the material structure changed. The calculated values of the *R.M.I.* parameter for Gly-POSS/PCL confirmed that the reduction in viscosity due to chain cleavage in the presence of Gly-POSS was smaller. 

These studies confirmed that polyhedral oligomeric silsesquioxane POSS, due to the presence of functional groups, can be used to modify the viscoelastic properties of poly(ε-caprolactone) at ambient and processing temperatures, and the relaxation behavior of melted PCL. Finally, the positive effect of Gly-POSS on the stability of the material during thermo-mechanical degradation is observed and provides potential for future studies. 

## Figures and Tables

**Table 1:** DSC analysis of PCL POSS samples.

Sample	T_g_, °C	T_m_, °C	T_c_, °C	*H_m_*, J g^−1^	*χ_c_*, % *
PCL	−70.93	56.90	32.63	65.10	48.5
PCL amine-POSS	−63.00	57.43	34.24	61.34	45.7
PCL HO-POSS	−63.33	56.37	31.55	68.39	50.9
PCL Gly-POSS	−62.35	58.49	30.44	54.92	40.9

T_g_—glass transition temperature, T_m_—melting temperature, T_c_—crystallization temperature, *H_m_*—enthalpy of melting, *χ_c_*—degree of crystallization. * the degree of crystallization was calculated according to the equation $\chi_{c}=\frac{\Delta H_{m}}{\Delta H_{m}^{0}}\times \frac{100}{w}$ [12,15] where $\Delta H_{m}^{0}$ is the enthalpy of melting of 100% crystalline PCL (138.5 J g^−1^ [12]), *w*—the weight fraction of material.

**Table 2:** TGA analysis of PCL POSS samples.

Sample	T_0.5%_, °C	T_1%_, °C	T_3%_, °C	T_5%_ °C	T_50%_, °C
PCL	238	328	382	388	421
PCL amine-POSS	196	319	376	385	424
PCL HO-POSS	160	259	373	385	424
PCL Gly-POSS	187	304	376	385	424

T_0.5,1,3,5,50%_—temperature at 0.5, 1, 3, 5, 50% weight loss.

## Data Availability

Not applicable.

## Supplementary Materials

The following supporting information can be downloaded at: https://www.mdpi.com/article/10.3390/polym14235078/s1, Figure S1: DSC plot for neat PCL; Figure S2: DSC plot for PCL amine-POSS; Figure S3: DSC plot for PCL HO-POSS; Figure S4: DSC plot for PCL Gly-POSS; Figure S5: Viscoelastic properties of molten PCL modified by the addition of POSS particles, complex viscosity *η**, storage shear modulus *G′*, loss shear modulus *G*″ at 100 °C as a function of angular frequency ω (rad⋅s^−1^), applied oscillation strain 1%; Table S1: Changes in cross points *G*′ = *G*″ determined from frequency sweep tests measured at 100 °C, oscillation shear rate 1% for samples thermally treated (100 °C, air flow) during 30, 60, 90, 120 min; Table S2: Values of relaxation modulus G_i_ (Pa) and relaxation times *λ_i_* (s) calculated using Maxwell models for melted PCL; Table S3: Values of relaxation modulus G_i_ (Pa) and relaxation times *λ_i_* (s) calculated using Maxwell models for melted PCL amine-POSS; Table S4: Values of relaxation modulus G_i_ (Pa) and relaxation times *λ_i_* (s) calculated using Maxwell models for melted PCL HO-POSS; Table S5: Values of relaxation modulus G_i_ (Pa) and relaxation times *λ_i_* (s) calculated using Maxwell models for melted PCL Gly-POSS.
