# Supplementary material for: The Effect of Various Polyhedral Oligomeric Silsesquioxanes on Viscoelastic, Thermal Properties and Crystallization of Poly(ε-caprolactone) Nanocomposites
Source: Polymers (Basel). 2022 Nov 23;14(23):5078. doi: 10.3390/polym14235078 (PMC9737336; doi:10.3390/polym14235078)
Supplement: Supplementary file 1 [file polymers-14-05078-s001.zip › polymers-2031328-supplementary.pdf]

The effect of polyhedral oligomeric silsesquioxanes on viscoelastic, thermal properties and crystallization of poly(ε-caprolactone) nanocomposites.

Magdalena Lipińska [1*]

[1] Lodz University of Technology; Institute of Polymer and Dye Technology
magdalena.lipinska@p.lodz.pl
* Correspondence: magdalena.lipinska@p.lodz.pl

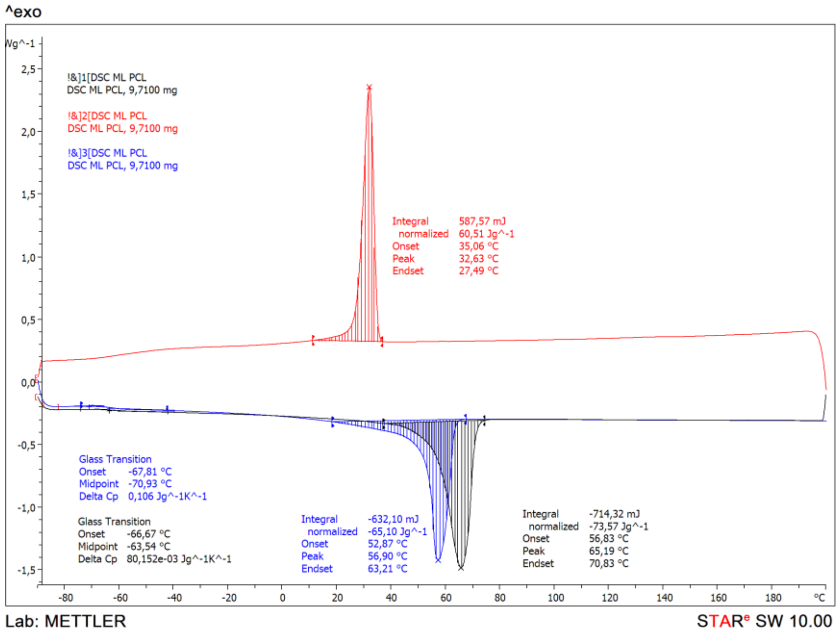

Figure S1: DSC plot for neat PCL.

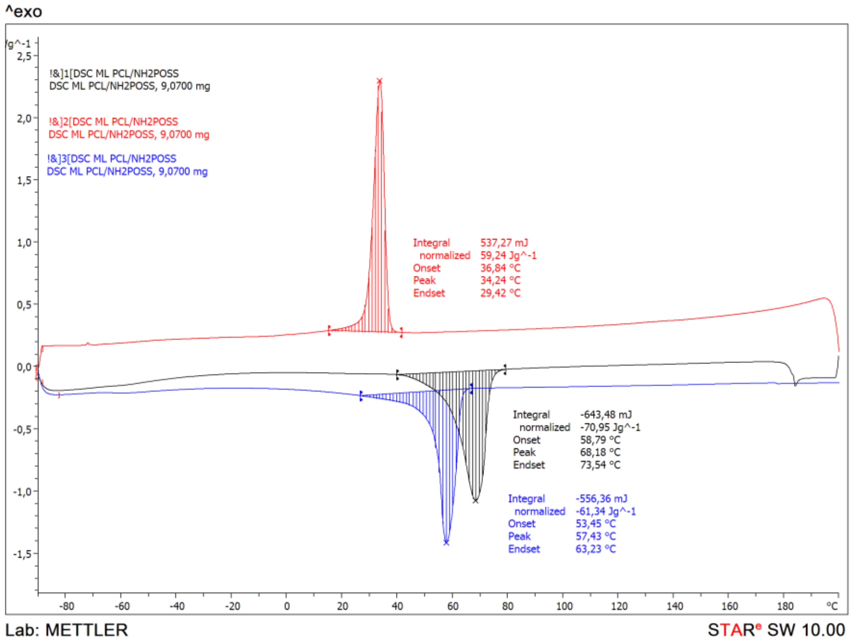

Figure S2: DSC plot for PCL amine-POSS.

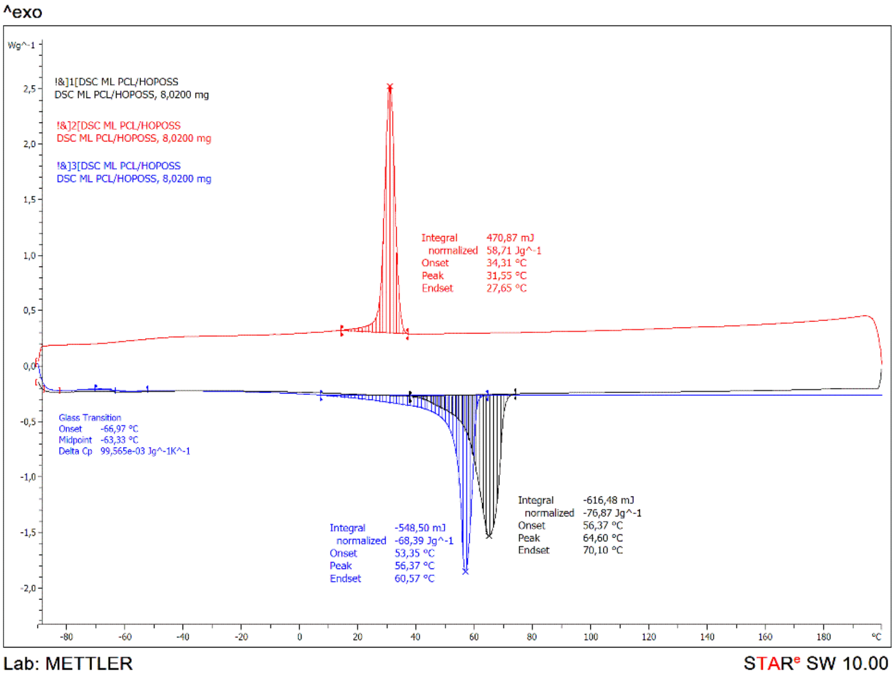

Figure S3: DSC plot for PCL HO-POSS

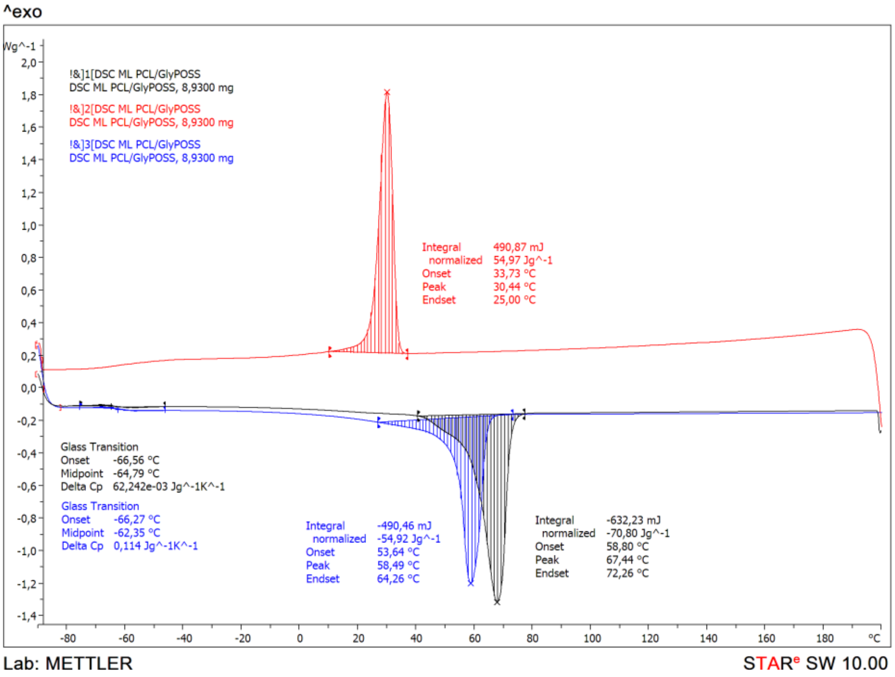

Figure S4: DSC plot for PCL Gly-POSS

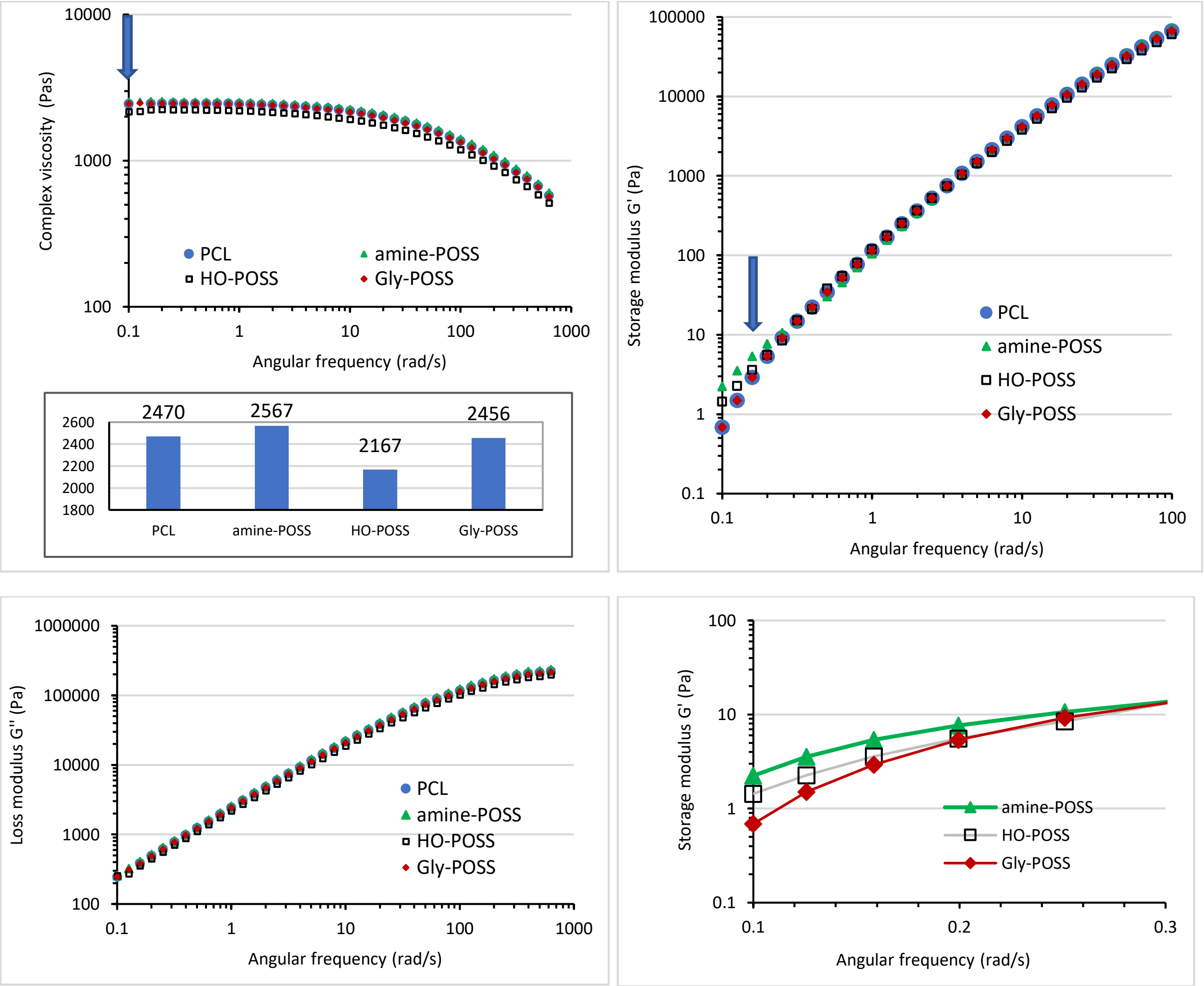

Figure S5: Viscoelastic properties of molten PCL modified by the addition of POSS particles, complex viscosity η^*, storage shear modulus G', loss shear modulus G'' at 100°C as a function of angular frequency ω ($rad{\cdot}s^{-1}$), applied oscillation strain 1%.

Table S1: Changes in cross points G′=G″ determined from frequency sweep tests measured at 100°C, oscillation shear rate 1% for samples thermally treated (100°C, air flow) during 30, 60, 90, 120 min.

100°C oxygen	G′ = G″ (kPa)	Angular frequency ω (rad·s^{-1})
PCL	200.20	350.84
30 min.	199.23	346.08
60 min.	198.10	346.08
90 min.	197.38	346.55
120 min.	196.54	346.17
PCL amine – POSS	212.70	354.81
30 min.	210.11	356.21
60 min.	208.91	359.62
90 min.	207.66	362.89
120 min.	207.01	365.37
PCL HO – POSS	176.27	354.74
30 min.	174.95	351.00
60 min.	173.65	350.23
90 min.	172.91	350.63
120 min.	172.03	349.84
PCL Gly – POSS	197.03	353.24
30 min.	194.67	347.53
60 min.	193.02	346.97
90 min.	192.16	347.79
120 min.	191.51	347.72

Table S2: Values of relaxation modulus G_i (Pa) and relaxation times λ_i (s) calculated using Maxwell models for melted PCL.

	PCL		PCL 30 min.		PCL 60 min.		PCL 120 min.	
n	Relaxation time λ (s)	Relaxation modulus G_i (Pa)	Relaxation time λ (s)	Relaxation modulus G_i (Pa)	Relaxation time λ (s)	Relaxation modulus G_i (Pa)	Relaxation time λ (s)	Relaxation modulus G_i (Pa)
1	$3.20\cdot10^{-4}$	542499	$2.81\cdot10^{-4}$	531081	$2.75\cdot10^{-4}$	526234	$2.56\cdot10^{-4}$	536416
2	$2.75\cdot10^{-3}$	252497	$2.38\cdot10^{-3}$	251440	$2.32\cdot10^{-4}$	249510	$2.30\cdot10^{-4}$	254032
3	0.01276	67025.5	$8.99\cdot10^{-3}$	77550.9	$8.37\cdot10^{-3}$	77212.5	$8.69\cdot10^{-3}$	78425.6
4	0.05386	9993.73	0.029048	20962.7	0.024881	23781.4	0.02782	21666.2
5	0.27599	661.473	0.103849	2900.61	0.07968	4208.63	0.0967	3160.47
6	1.79548	18.3076	0.439952	268.218	0.319187	463.326	0.403732	300.645
R^2	0.9996		0.9999		0.9999		0.9999	

Table S3: Values of relaxation modulus G_i (Pa) and relaxation times λ_i (s) calculated using Maxwell models for melted PCL amine-POSS.

	PCL amine-POSS		PCL amine-POSS 30 min.		PCL amine-POSS 60 min.		PCL amine-POSS 120 min.	
n	Relaxation time λ (s)	Relaxation modulus G_i (Pa)	Relaxation time λ (s)	Relaxation modulus G_i (Pa)	Relaxation time λ (s)	Relaxation modulus G_i (Pa)	Relaxation time λ (s)	Relaxation modulus G_i (Pa)
1	$3.62\cdot10^{-4}$	523724	$2.11\cdot10^{-4}$	645527	$2.56\cdot10^{-4}$	554894	$3.34\cdot10^{-4}$	480866
2	$2.82\cdot10^{-3}$	265872	$2.20\cdot10^{-3}$	273195	$2.25\cdot10^{-4}$	271005	$2.43\cdot10^{-4}$	259637
3	0.013317	67435.3	$7.75\cdot10^{-3}$	80540.6	$8.56\cdot10^{-4}$	80616.9	$9.99\cdot10^{-4}$	74825.4
4	0.058237	9218.51	0.021902	26771.7	0.027032	21806.9	0.035806	15836.9
5	0.315852	558.908	0.06372	5586.05	0.088753	3280.98	0.150764	1562.18
6	4.6678	7.41024	0.229386	769.591	0.336022	386.964	0.781878	79.561
R^2	0.9999		0.9999		0.9999		0.9999	

Table S4: Values of relaxation modulus G_i (Pa) and relaxation times λ_i (s) calculated using Maxwell models for melted PCL HO-POSS.

	PCL HO-POSS		PCL HO-POSS 30 min.		PCL HO-POSS 60 min.		PCL HO-POSS 120 min.	
n	Relaxation time λ (s)	Relaxation modulus G_i (Pa)	Relaxation time λ (s)	Relaxation modulus G_i (Pa)	Relaxation time λ (s)	Relaxation modulus G_i (Pa)	Relaxation time λ (s)	Relaxation modulus G_i (Pa)
1	$3.51\cdot10^{-4}$	418478	$4.60\cdot10^{-4}$	360262	$4.62\cdot10^{-4}$	352363	$4.85\cdot10^{-4}$	337305
2	$2.47\cdot10^{-3}$	216847	$2.57\cdot10^{-3}$	200211	$2.55\cdot10^{-3}$	199451	$2.54\cdot10^{-3}$	195898
3	$9.98E\cdot10^{-3}$	66093.4	$9.87\cdot10^{-3}$	65163.5	$9.67\cdot10^{-3}$	64951.1	$9.55\cdot10^{-3}$	64513.2
4	0.035379	14301.9	0.033534	15231.6	0.032137	15688.6	0.031527	15984.5
5	0.143364	1789.7	0.129298	1898.81	0.119075	2090.52	0.11799	2131.34
6	0.612703	217.027	0.52232	294.669	0.459565	335.106	0.502091	327.167
R^2	0.9999		0.9999		0.9999		0.9999	

Table S5: Values of relaxation modulus G_i (Pa) and relaxation times λ_i (s) calculated using Maxwell models for melted PCL Gly-POSS.

	PCL Gly-POSS		PCL Gly-POSS 30 min.		PCL Gly-POSS 60 min.		PCL Gly-POSS 120 min.	
n	Relaxation time λ (s)	Relaxation modulus G_i (Pa)	Relaxation time λ (s)	Relaxation modulus G_i (Pa)	Relaxation time λ (s)	Relaxation modulus G_i (Pa)	Relaxation time λ (s)	Relaxation modulus G_i (Pa)
1	$1.59\cdot10^{-4}$	808345	$2.50\cdot10^{-4}$	532103	$2.70\cdot10^{-4}$	498055	$5.24\cdot10^{-4}$	357270
2	$2.31\cdot10^{-3}$	264878	$2.29\cdot10^{-3}$	255195	$2.30\cdot10^{-3}$	250937	$2.59\cdot10^{-3}$	216388
3	$8.03\cdot10^{-3}$	59280.6	$8.94\cdot10^{-3}$	78052.8	$8.87\cdot10^{-3}$	77138.2	$9.74\cdot10^{-3}$	70963.8
4	0.016784	28875.1	0.029935	20034.5	0.029482	20246.6	0.032526	17430
5	0.05257	9428.75	0.109752	2695.23	0.110741	2766.16	0.121829	2284.66
6	0.285493	878.338	0.401028	315.805	0.465942	294.516	0.482851	262.717
R^2	0.9999		0.9999		0.9999		0.9999	
